# Postoperative insulin requirements in surgical patients receiving fully automated insulin delivery in the hospital

**DOI:** 10.1111/dom.70170

**Published:** 2025-10-06

**Authors:** Gabija Krutkyte, Clara Escorihuela‐Altaba, Michael S. Hughes, Christos T. Nakas, David Herzig, Lia Bally

**Affiliations:** ^1^ Department of Diabetes, Endocrinology, Nutritional Medicine and Metabolism Inselspital, Bern University Hospital, University of Bern Bern Switzerland; ^2^ Department of Anaesthesiology and Pain Medicine lnselspital, Bern University Hospital, University of Bern Bern Switzerland; ^3^ Graduate School for Cellular and Biomedical Sciences University of Bern Bern Switzerland; ^4^ Diabetes Center Berne Bern Switzerland; ^5^ Division of Endocrinology, Metabolism, and Lipids, Department of Medicine Emory University School of Medicine Atlanta Georgia USA; ^6^ School of Agricultural Sciences, Laboratory of Biometry University of Thessaly Volos Greece; ^7^ Department of Clinical Chemistry Inselspital, Bern University Hospital, University of Bern Bern Switzerland

**Keywords:** clinical physiology, CSII, glycaemic control, insulin pump therapy, insulin therapy

## 1 BACKGROUND

Surgery‐induced metabolic stress, immobilisation and adjunctive therapies (e.g., glucocorticoids, nutritional support) cause dynamic changes in insulin requirements.^1^, ^2^ A mismatch between prescribed and actual insulin needs predisposes patients to dysglycaemia, which is associated with prolonged hospitalisation and adverse outcomes.^3^, ^4^, ^5^ Fully automated insulin delivery (AID) systems continuously titrate insulin to maintain target glucose levels, offering both effective perioperative support for complex patients^6^, ^7^ and insights into insulin requirements. This study examined perioperative insulin delivery in patients with fully AID and identified determinants of insulin needs to inform postoperative glycaemic strategies.

## 2 METHODS

### 2.1 Study design

Data from two randomised controlled trials (NCT05392452 and NCT04361799) investigating the glycaemic efficacy of fully AID in hospitalised surgical patients with insulin‐requiring non‐type 1 diabetes were pooled and retrospectively analysed. Both trials had ethics approval (2020‐01024, 2022‐D0034) and all participants provided written informed consent. Eligible adults underwent elective surgery, were expected to require insulin, and remained hospitalised ≥72 h postoperatively; type 1 diabetes was excluded. The AID system comprised the Dexcom G6 subcutaneous continuous glucose monitoring (Dexcom, USA), an android smartphone hosting the CamAPS HX application with a model predictive control algorithm (University of Cambridge, Cambridge, UK), and a subcutaneous insulin pump—either YpsoPump (Ypsomed AG, Burgdorf, Switzerland) or the DANA RS insulin pump (Diabecare, Sooil, Seoul, South Korea), delivering fast‐acting insulin aspart (Fiasp, Novo Nordisk). AID was initialised using participants' body weight and estimated total daily insulin dose (TDD), with a glucose target of 5.8 mmol/L.

### 2.2 Data collection

Continuous insulin delivery and glucose data were retrieved from a cloud‐based diabetes management platform (Glooko, Inc., Palo Alto, California, USA). Clinical data, including nutrition support type (parenteral, enteral or both) and glucocorticoid use (documented daily as a binary variable), were extracted from electronic health records. For a subset of patients, daily carbohydrate (CHO) intake (oral plus nutritional support, calculated over 24‐h periods [00:00 to 23:59]) was obtained from the hospital's electronic meal management software.

### 2.3 Statistical analysis

TDD and weight‐normalised TDD (TDD/kg) were calculated for each patient, from the first postoperative day (00:00) until the 10th postoperative day or discharge, excluding the day of surgery and discharge. Day‐to‐day variability was assessed by the coefficient of variation (CV) of TDD, calculated for each patient.

Changes in TDD were analysed with a linear mixed‐effects model. Predictor significance was tested with Type III Wald chi‐square tests, and model assumptions were verified graphically.

Linear mixed‐effects models assessed effects of postoperative day, nutrition support (parenteral vs. enteral vs. concomitantly administered parenteral and enteral) and glucocorticoid therapy on TDD/kg, adjusted for surgery type, glycated haemoglobin (HbA1c), sex, age and body mass index (BMI). Postoperative day was included as a fixed effect and participant as a random intercept to account for within‐subject correlation. Non‐significant terms were removed to reduce model complexity and improve interpretability. For patients with CHO data, a quadratic term accounted for non‐linear effects.

Results from the fixed‐effects estimates are presented with 95% confidence intervals [95% CI], *p*‐values, and using Forest plots, while non‐linear effects of CHO supply on TDD/kg are visualised graphically. Descriptive statistics are presented as mean ± standard deviation (SD) for normally distributed variables or median [25th; 75th percentile] for non‐normally distributed variables unless specified otherwise. Analyses were performed using R (4.4.1).

## 3 RESULTS

### 3.1 Clinical context and glucose control

Thirty‐six patients (277 patient‐days, mean age 67.8 ± 11 years, 39% female, HbA1c 7.2% [6.4; 8]) were analysed (Table S1). Abdominal surgery was most common (*n* = 27 (75%), including seven total pancreatectomies). Postoperatively, 36% received parenteral nutrition, 8% enteral and 14% parenteral and enteral nutrition concomitantly. Postoperative glucocorticoids were prescribed in 14% of patients. In a subset of 13 patients (99 patient‐days) for whom detailed CHO data were available, the mean between‐participant daily CHO supply was 163 ± 42 g (105 ± 68 g with solely oral nutrition; 123 g [81; 167] with enteral nutrition support [as a stand‐alone or in combination with oral nutrition]; 187 g [170; 223] with parenteral nutrition [with or without accompanying oral nutrition]). The concomitant use of parenteral with enteral nutrition delivered the highest quantities of 286 [264; 302] g/day.

**FIGURE 1:**
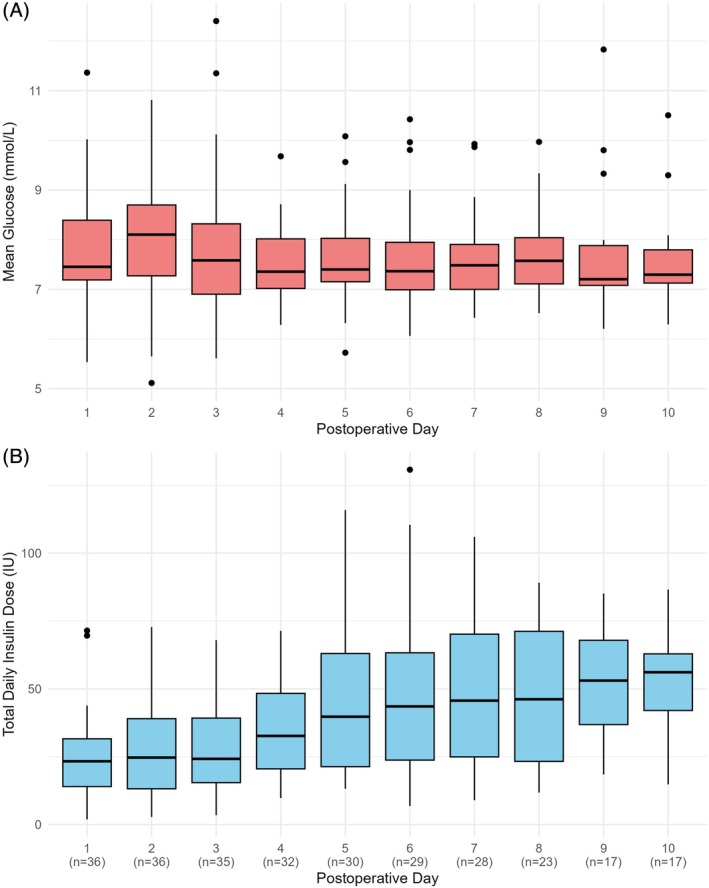
Postoperative mean sensor daily glucose and total daily insulin dose. (A) Time course of mean daily sensor glucose following surgery. (B) Time course of total daily insulin dose following surgery. Boxplots show median (solid line), interquartile range (IQR; box outline) and spread of data points without outliers (whiskers). Outliers are defined as measurements beyond 1.5 × IQR (symbols). *n* represents the number of observations available on the corresponding day.

Postoperative mean daily glucose levels were maintained between 7.2 and 8.1 mmol/L, and time with glucose levels between 3.9 and 10.0 mmol/L was 89.1% [82.2; 93.2] (Figure 1A, Table S2).

### 3.2 Insulin requirements

Figure 1B illustrates the temporal evolution of postoperative insulin requirements, with daily doses normalised by body weight presented in Table S3. Insulin requirements were lowest on the first postoperative day and increased over time after surgery, peaking on Day 10 at 0.65 [0.47; 0.81] units/kg, representing a 2.41 [2.08; 3.36]‐fold change. Between‐day variability in insulin requirements during the 10 postoperative days was substantial (CV 42 ± 18.7%).

### 3.3 Determinants of insulin requirements

**FIGURE 2:**
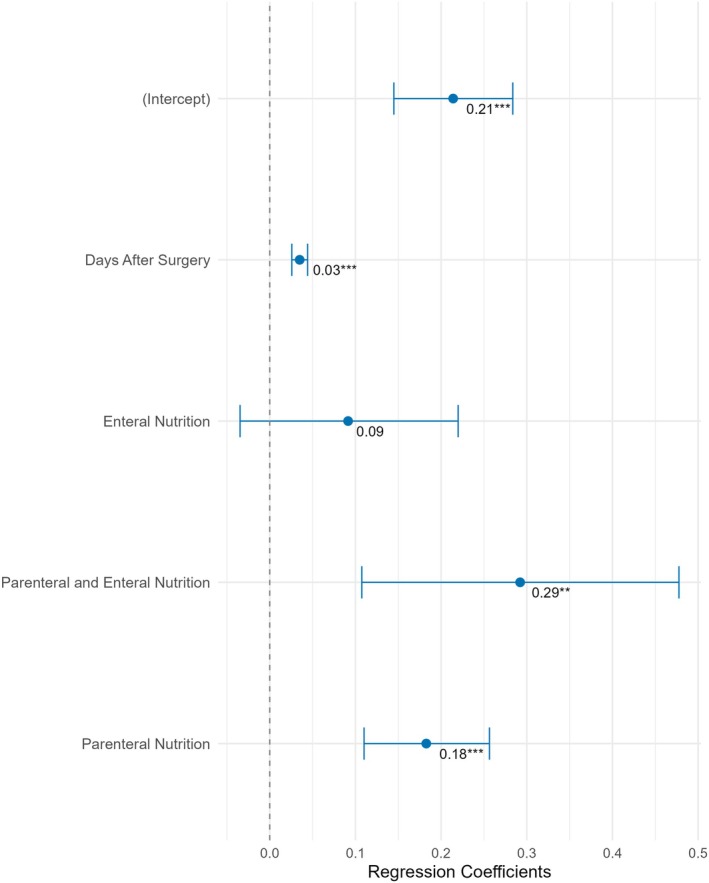
Forest plot of factors predicting total daily insulin dose normalised for body weight (total daily dose [TDD]/kg). The forest plot illustrates the regression coefficients (with 95% confidence intervals) of key predictors on TDD/kg. Predictors include days after surgery and nutrition support type (enteral, parenteral and combined), while intercept represents the estimated TDD/kg on days with oral nutrition only. Significant predictors are marked with asterisks (**p [0.001; 0.01], ***p < 0.001).

Parenteral nutrition, concomitant parenteral and enteral nutrition and days after surgery were significantly associated with higher daily insulin requirements when compared to days with oral nutrition only. TDD/kg was 0.18 IU/kg [0.1; 0.3] higher on days with parenteral nutrition and 0.03 IU/kg [0.02; 0.04] higher with each day after surgery (all *p* < 0.001). Preoperative HbA1c, BMI, age, sex, pancreatic surgery, postoperative enteral nutrition and glucocorticoid therapy were not significant predictors. Regression coefficients for daily insulin dose are presented in Figure 2.

In patients with CHO data, insulin requirements increased non‐linearly with CHO intake, from 0.45 IU/kg (fasting) to 0.95 IU/kg at 300 g/day (*p* < 0.001; Figure S1).

## 4 CONCLUSIONS

This study characterised daily postoperative insulin delivery and its determinants in surgery patients using fully AID. Excellent glucose control (89.1% time in range) enabled robust insights into postoperative insulin needs, which varied substantially—lowest immediately after surgery and rising 2.4‐fold by Day 10, with a day‐to‐day CV of 42%. Parenteral nutrition, with or without enteral supplementation, was a significant predictor of insulin needs, alongside postoperative day. CHO supply exhibited a non‐linear positive association with insulin dose, although the dose–response may be influenced by unmeasured factors such as feeding schedule, nutrient composition and individual insulin sensitivity. Importantly, postoperative day independently predicted insulin requirements even after accounting for CHO intake, indicating additional physiological mechanisms.

Day‐to‐day insulin variability in this cohort (42 ± 18.7%) significantly exceeded that of non‐surgical inpatients using identical AID algorithms (30 ± 16%),^8^ highlighting the unique challenges of the immediate postoperative period and the necessity for vigilant titration. Consistent with prior evidence,^8^ baseline characteristics (BMI, age, sex and HbA1c) were not predictive. These findings suggest that insulin therapy cannot rely on admission parameters alone, but rather must be dynamically adjusted to recovery and interventions, particularly nutrition. The lack of a statistically significant effect of glucocorticoid therapy may be attributable to both the limited sample size of treated patients and the binary categorisation of glucocorticoid use, which precluded dose‐dependent analysis. This is a crucial area for future research in larger studies.

Parenteral nutrition emerged as a strong predictor of insulin requirements. This observation is physiologically consistent, as intravenous glucose administration bypasses the incretin effect that normally potentiates glucose‐stimulated insulin secretion during oral or enteral nutrient absorption.^9^ Combined parenteral‐enteral nutrition generated the highest insulin requirements, as its CHO load often exceeded 200 g/day—the recommended threshold for hospitalised patients with diabetes.^10^ These findings suggest that careful moderation of carbohydrate intake is a practical way to support postoperative glycaemic control, especially where automated titration is unavailable.

Limitations include a small sample size (36 patients), a post hoc approach, potential underestimation of endogenous insulin and that carbohydrate data were available for only a subset of patients, restricting the scope of the dose–response relationship between nutrition and insulin needs. Data provision from a single hospital may limit external validity. Findings may not generalise beyond surgical populations or less invasive procedures.

In conclusion, fully AID provides valuable insights into metabolic dynamics. The highly variable postoperative insulin requirements, driven by nutrition support and carbohydrate provision, underscore the need for adaptive glucose management. While our hypothesis‐generating study robustly characterises this phenomenon, larger prospective trials are needed to validate these predictors and develop adaptive AID‐dosing algorithms that account for nutritional variability and other challenges to optimise postoperative care.

## AUTHOR CONTRIBUTIONS

LB and DH designed the study. GK, LB and DH coordinated study procedures and managed ethics approval. GK, CE‐A, MSH, CTN and DH contributed to data analysis, including statistical analyses. All authors contributed to the interpretation of the results, critically revised the article and approved the final manuscript. All authors made the decision to submit for publication. LB is the guarantor of this work and, as such, had full access to all the data in the study and takes responsibility for the integrity of the data and the accuracy of the data analysis.

## CONFLICT OF INTEREST STATEMENT

Lia Bally reports having received speaker and advisory board honoraria from Dexcom, Inc., Novo Nordisk and Ypsomed. David Herzig reports having received speaker honoraria from Ypsomed. Michael S. Hughes reports having consulted for Dexcom, Inc. For the remaining authors, none were declared.

## Supporting information


**Table S1.** Clinical context.
**Table S2.** CGM metrics and insulin delivery during hospitalisation.
**Table S3.** Postoperative total daily insulin dose normalised by body weight.
**Figure S1.** Effect of carbohydrate intake on total daily insulin dose normalised for body weight (IU/kg).

## Data Availability

The data that support the findings of this study are available from the corresponding author upon reasonable request.
