# Supplementary material for: Postoperative insulin requirements in surgical patients receiving fully automated insulin delivery in the hospital
Source: Diabetes Obes Metab. 2025 Oct 6;28(1):741–5. doi: 10.1111/dom.70170 (PMC12622854; doi:10.1111/dom.70170)
Supplement: Supplementary file 1 — Table S1. Clinical context. Table S2. CGM metrics and insulin delivery during hospitalisation. Table S3. Postoperative total daily insulin dose normalised by body weight. Figure S1. Effect of carbohydrate intake on total daily insulin dose normalised for body weight (IU/kg). [file DOM-28-741-s001.docx]

| **Table S1. Clinical Context** | | | | |
| --- | --- | --- | --- | --- |
|  |  |  | **N = 36** | |
| **Patient Population** | | |  |  |
| Age (years) | | | 67.8 ± 11 | |
| Biological sex (female) | | | 14 (38.9) | |
| BMI (kg/m²) | | | 29.7 ± 6.5 | |
| HbA1c (%) | | | 7.2 [6.4; 8] | |
| Patients receiving insulin therapy at baseline | | | 19 (52.8) | |
| Insulin total daily dose at baseline (IU/d)^†^ | | | 25 [18.5; 50] | |
| **Surgery Details** | | |  | |
| Type of surgery | | |  | |
| Abdominal | | | 27 (75) | |
| Pancreatic resection | | | 18 (50) | |
| Vascular | | | 4 (11.1) | |
| Orthopaedic | | | 3 (8.3) | |
| Neurosurgery | | | 1 (2.8) | |
| Thoracic | | | 1 (2.8) | |
| Duration (hours) | | | 3.9 [2.8; 6] | |
| Intraoperative glucocorticoid administration**^‡^** | | | 21 (58.3) | |
| **Postoperative Care** | | |  | |
| Patients receiving | | |  | |
| Parenteral nutrition | | | 13 (36.1) | |
| Enteral nutrition | | | 3 (8.3) | |
| Parenteral and enteral nutrition | | | 5 (13.9) | |
| Glucocorticoid administration | | | 5 (13.9) |  |
| Total daily carbohydrate supply (g)^§^ | | | 163.2 (42) |  |
| Length of stay | | | 11.5 [7.8; 21] | |
| Data are mean±SD, median [25th; 75th percentile] or n (%). *BMI, body-mass-index.*  ^†^ Includes only the patients who were on insulin prior to enrolment  **^‡^** Intraoperative glucocorticoids were given as a single dose of 4mg (n=9) or 8mg (n=12) IV dexamethasone once at anesthesia induction  § Based on the data subset of 13 patients and 99 observed days | | | | |

| **Table S2. CGM Metrics And Insulin Delivery During Hospitalization** | |
| --- | --- |
| Proportion of time spent at glucose concentration |  |
| 3.9-10.0 mmol/L (%) | 89.1 [82.2; 93.2] |
| >10.0 mmol/L (%) | 10.2 [6.5; 16.8] |
| >20.0 mmol/L (%) | 0.0 [0.0; 0.0] |
| <3.9 mmol/L (%) | 0.3 [0.0; 0.8] |
| <3.0 mmol/L (%) | 0.0 [0.0; 0.2] |
| Mean glucose concentration (mmol/L) | 7.7 ± 0.6 |
| SD glucose concentration (mmol/L) | 1.9 ± 0.5 |
| CV of glucose concentration (%) | 24.9 ± 4.9 |

Data are mean±SD or median [25th; 75th percentile].

*CGM, continuous glucose monitoring; SD, standard deviation; CV, coefficient of variation; IU, international units.*

| **Table S3. Postoperative Total Daily Insulin Dose Normalized by Body Weight** | |
| --- | --- |
| Postoperative day | Insulin dose (IU/kg) |
| 1 | 0.27 [0.14; 0.39] |
| 2 | 0.3 [0.17; 0.4] |
| 3 | 0.29 [0.22; 0.41] |
| 4 | 0.38 [0.28; 0.53] |
| 5 | 0.46 [0.27; 0.78] |
| 6 | 0.51 [0.32; 0.93] |
| 7 | 0.49 [0.35; 0.86] |
| 8 | 0.59 [0.34; 0.81] |
| 9 | 0.61 [0.41; 0.77] |
| 10 | 0.65 [0.47; 0.81] |

Data are median [25th; 75th percentile].

**Figure S1. Effect of Carbohydrate Intake on Total Daily Insulin Dose Normalized For Body Weight (IU/kg).**


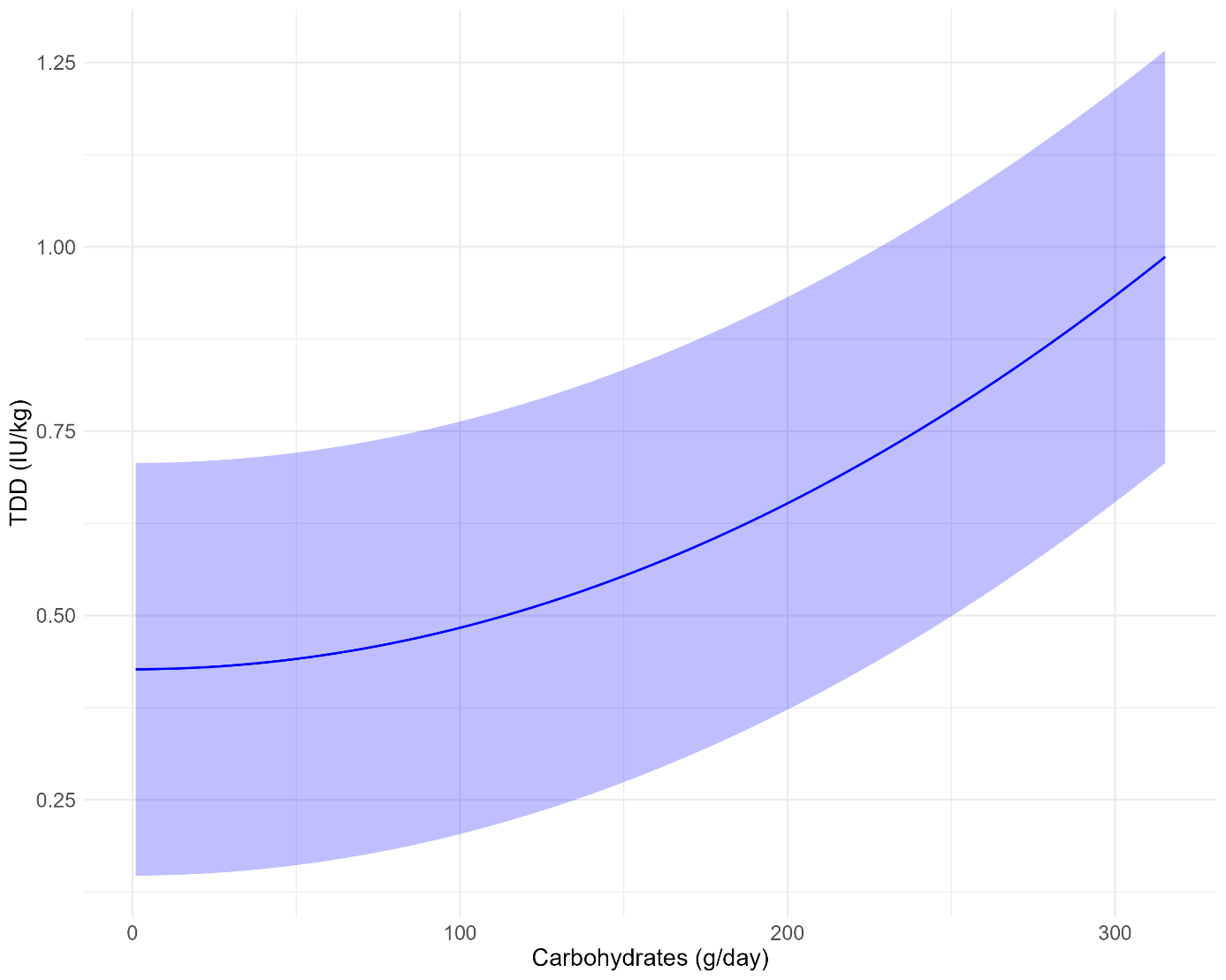


The figure illustrates the relationship between daily carbohydrate intake and TDD/kg (IU/kg) in the postoperative period. The solid blue line represents the estimated TDD/kg, while the shaded area indicates the 95% confidence interval.

*TDD, total daily insulin dose; IU, international units.*
