# Supplementary material for: Postoperative insulin requirements in surgical patients receiving fully automated insulin delivery in the hospital
Source: Diabetes Obes Metab. Author manuscript; Available in PMC 2025 Nov 18. (PMC12622854; doi:10.1111/dom.70170)
Supplement: Supplementary file 1 [file NIHMS2116784-supplement-1.pdf]

Table S1. Clinical Context

	N = 36
**Patient Population**	
Age (years)	67.8 ± 11
Biological sex (female)	14 (38.9)
BMI (kg/m²)	29.7 ± 6.5
HbA1c (%)	7.2 [6.4; 8]
Patients receiving insulin therapy at baseline	19 (52.8)
Insulin total daily dose at baseline (IU/d)†	25 [18.5; 50]
**Surgery Details**	
Type of surgery	
Abdominal	27 (75)
Pancreatic resection	18 (50)
Vascular	4 (11.1)
Orthopaedic	3 (8.3)
Neurosurgery	1 (2.8)
Thoracic	1 (2.8)
Duration (hours)	3.9 [2.8; 6]
Intraoperative glucocorticoid administration‡	21 (58.3)
**Postoperative Care**	
Patients receiving	
Parenteral nutrition	13 (36.1)
Enteral nutrition	3 (8.3)
Parenteral and enteral nutrition	5 (13.9)
Glucocorticoid administration	5 (13.9)
Total daily carbohydrate supply (g)§	163.2 (42)
Length of stay	11.5 [7.8; 21]

Data are mean±SD, median [25th; 75th percentile] or n (%). *BMI, body-mass-index.*
† Includes only the patients who were on insulin prior to enrolment
‡ Intraoperative glucocorticoids were given as a single dose of 4mg (n=9) or 8mg (n=12) IV dexamethasone once at anesthesia induction
§ Based on the data subset of 13 patients and 99 observed days

Table S2. CGM Metrics And Insulin Delivery During Hospitalization

Proportion of time spent at glucose concentration	
3.9-10.0 mmol/L (%)	89.1 [82.2; 93.2]
>10.0 mmol/L (%)	10.2 [6.5; 16.8]
>20.0 mmol/L (%)	0.0 [0.0; 0.0]
<3.9 mmol/L (%)	0.3 [0.0; 0.8]
<3.0 mmol/L (%)	0.0 [0.0; 0.2]
Mean glucose concentration (mmol/L)	7.7 ± 0.6
SD glucose concentration (mmol/L)	1.9 ± 0.5
CV of glucose concentration (%)	24.9 ± 4.9

Data are mean±SD or median [25th; 75th percentile].
CGM, continuous glucose monitoring; SD, standard deviation; CV, coefficient of variation; IU, international units.

Table S3. Postoperative Total Daily Insulin Dose Normalized by Body Weight

Postoperative day	Insulin dose (IU/kg)
1	0.27 [0.14; 0.39]
2	0.3 [0.17; 0.4]
3	0.29 [0.22; 0.41]
4	0.38 [0.28; 0.53]
5	0.46 [0.27; 0.78]
6	0.51 [0.32; 0.93]
7	0.49 [0.35; 0.86]
8	0.59 [0.34; 0.81]
9	0.61 [0.41; 0.77]
10	0.65 [0.47; 0.81]

Data are median [25th; 75th percentile].

Figure S1. Effect of Carbohydrate Intake on Total Daily Insulin Dose Normalized For Body Weight (IU/kg).

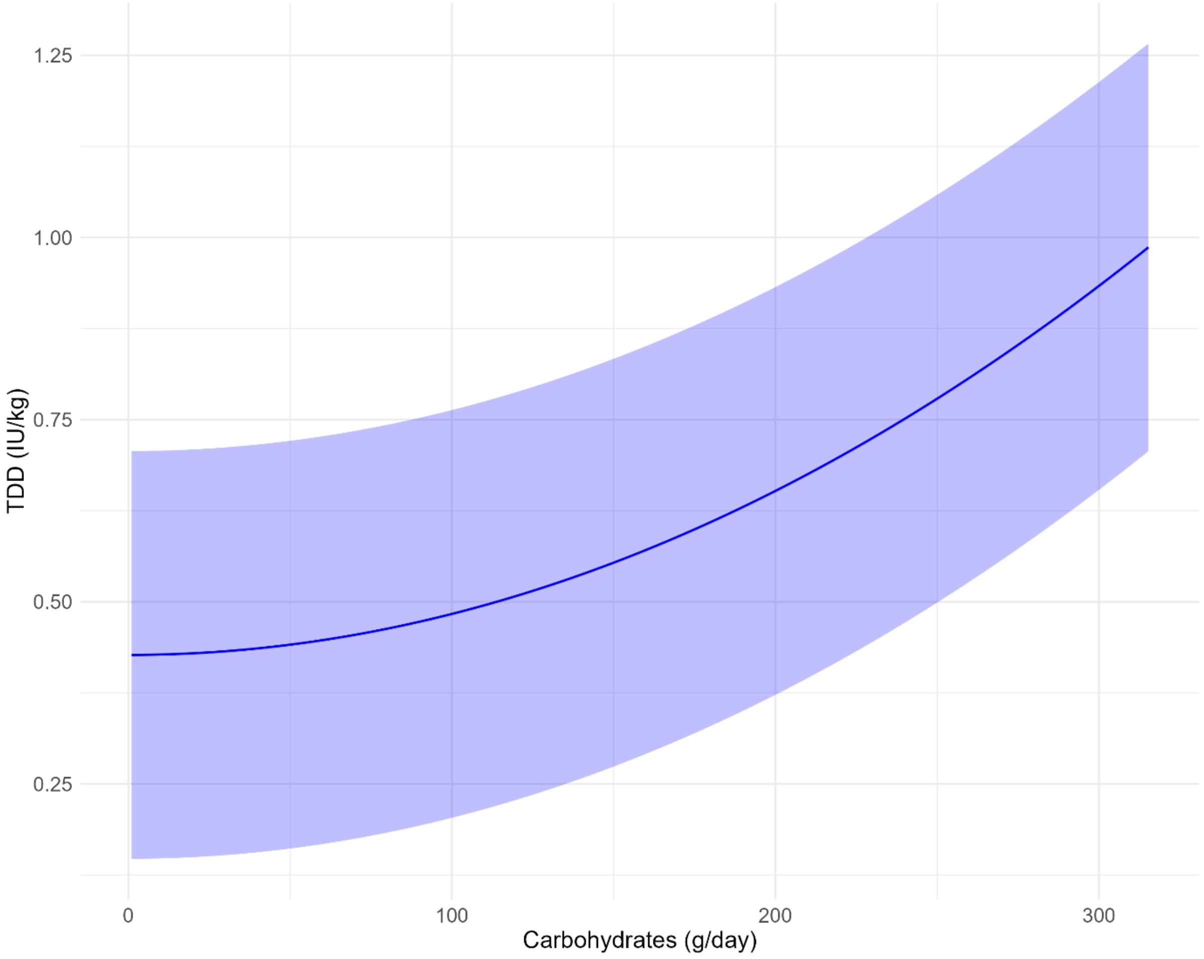

The figure illustrates the relationship between daily carbohydrate intake and TDD/kg (IU/kg) in the postoperative period. The solid blue line represents the estimated TDD/kg, while the shaded area indicates the 95% confidence interval.

TDD, total daily insulin dose; IU, international units.
